# A retrospective study of the prevalence of the canine degenerative myelopathy associated superoxide dismutase 1 mutation (*SOD1:c.118G > A*) in a referral population of German Shepherd dogs from the UK

**DOI:** 10.1186/2052-6687-1-10

**Published:** 2014-09-25

**Authors:** Angela L Holder, James A Price, Jamie P Adams, Holger A Volk, Brian Catchpole

**Affiliations:** Department of Pathology and Pathogen Biology, Royal Veterinary College, Hawkshead Lane, North Mymms, Hatfield, Hertfordshire, AL9 7TA UK; Department of Clinical Sciences and Services, Royal Veterinary College, Hawkshead Lane, North Mymms, Hatfield, Hertfordshire, AL9 7TA UK; Boehringer Ingelheim Ltd, Ellesfield Avenue, Bracknell, Berkshire, RG12 8YS UK

**Keywords:** Ataxia, Degenerative myelopathy, Genetic test, German shepherd dog, Superoxide dismutase 1

## Abstract

**Background:**

Canine degenerative myelopathy (CDM) is an adult onset, progressive neurodegenerative disease of the spinal cord. The disease was originally described in the German Shepherd dog (GSD), but it is now known to occur in many other dog breeds. A previous study has identified a mutation in the superoxide dismutase 1 gene (*SOD1:c.118G > A*) that is associated with susceptibility to CDM. In the present study, restriction fragment length polymorphism (RFLP) analysis was used to genotype GSD for *SOD1:c.118G > A* in order to estimate the prevalence of the mutation in a referral population of GSD in the UK.

**Results:**

This study demonstrated that the RFLP assay, based on use of PCR and subsequent digestion with the Eco571 enzyme, provided a simple genotyping test for the *SOD1:c.118G > A* mutation. In a young GSD population (i.e. dogs less than 6 years of age, before clinical signs of the disease usually become apparent), 8 of 50 dogs were found to be homozygous and a further 19 were heterozygous for the mutation. In dogs over 8 years of age, 21 of 50 dogs admitted to a tertiary referral hospital with pelvic limb ataxia as a major clinical sign were homozygous for the mutation, compared to none of 50 dogs of similar age, but where no neurological disease was reported on referral.

**Conclusions:**

This data suggests that genotyping for the *SOD1:c.118G > A* mutation is clinically applicable and that the mutation has a high degree of penetrance. Genotyping might also be useful for screening the GSD population to avoid mating of two carriers, but since the allele frequency is relatively high in the UK population of GSD, care should be taken to avoid reduction in genetic diversity within the breed.

## Lay summary

Canine degenerative myelopathy (CDM) is an adult onset, progressive disease where the spinal nerve cord degenerates.

This disease occurs in several dog breeds, but German shepherd dogs (GSD) are particularly susceptible. A previous study identified a genetic change (mutation) in the superoxide dismutase 1 gene (SOD1) that is associated with susceptibility to CDM.

Protein sequences in the body are made up of different types of building blocks called amino acids. Each type of amino acid is encoded by particular DNA code. The mutation, called SOD1:c.118G>A, encodes a change in the SOD1 protein (amino acid position 118 of the protein), where the usual amino acid glycine (G) has been replaced by the amino acid alanine (A), thus changing the structure and possible function of the protein.

In this study, a simple molecular DNA “finger print” type assay (called restriction fragment length polymorphism, RFLP) was used to genotype German shepherd dogs for the SOD1 mutation to estimate how frequently it occurs in this breed in the UK.

This study demonstrated that the genetic assay provided a simple test for the SOD1 mutation. In a young GSD population (i.e. dogs less than six years of age, before clinical signs of the disease usually become apparent), 8 of 50 dogs were found to carry two copies of the mutation (homozygous), a further 19 carried one copy (heterozygous for the mutation), while the remainder had no copies, (homozygous normal). In GSD dogs over eight years of age, 21 of 50 dogs admitted to a tertiary referral hospital with clinical signs of neurodegeneration (pelvic limb ataxia) were homozygous for the mutation, and 29 were heterozygous. In contrast, none of 50 GSD dogs of similar age, with no reported neurological disease were homozygous for the mutation, while 26 were heterozygous.

This study suggests that genotyping for the SOD1 mutation may be clinically useful. Genotyping might also be used for screening the GSD population to avoid mating of two carriers. However, because the frequency of the mutation is relatively high in the UK population of GSD, extreme care should be taken to avoid reduction in genetic diversity within the breed.

## Background

Canine degenerative myelopathy (CDM) is a progressive neurodegenerative disease of the spinal cord. The disease was originally described in the German Shepherd dog (GSD) [1], where it is a main cause of premature mortality [2], but it has since been shown to occur in many other dog breeds [3]. The onset of clinical signs typically occurs from around 8 years of age, with dogs showing asymmetric progressive upper motor neuron paraparesis and ataxia of the pelvic limbs, worn nails and a lack of spinal pain upon palpation of the vertebral column. Paraparesis progresses to lower motor neuron paraplegia within 9 to 18 months, usually necessitating euthanasia [3]. However, if euthanasia is delayed, as is possible in smaller breeds where care at home can be given for longer, clinical signs can progress to the thoracic limbs [4]. Post-mortem histology is most compatible with a primary central axonopathy of the spinal cord. Axons and associated myelin degenerate in a segmental pattern and affect all funiculi and involve mainly general proprioceptive sensory, somatic sensory and motor tracts, but lack observable neuronal cell body degeneration or loss [5, 6]. Diagnosis of CDM is not straightforward, since similar clinical signs are seen with other neurological diseases, such as intervertebral disc or lumbosacral disease. There is no definitive ante-mortem diagnostic test for CDM and the diagnosis is usually based on exclusion of other neurological diseases [3], although co-morbidities can be present.

Similarities have been observed between CDM and the motor neurone disease, amyotrophic lateral sclerosis (ALS) in humans. ALS is a disease typically seen between the ages of 45 and 60, where degeneration of the upper and lower motor neurons culminates in paralysis and death [7]. Genetic studies in familial ALS identified an association between the disease and mutations in the gene encoding superoxide dismutase 1 (SOD1) [8]. SOD1 is an enzyme involved in scavenging of superoxide free radicals within cells and thus plays an important role in prevention of oxidative damage. Studies suggest enzyme activity is unaffected by the presence of SOD1 mutations; instead proteins encoded by the mutated version of the gene demonstrate an increased propensity to form aggregates [9, 10], although it has yet to be established how this relates to the pathogenesis of neuronal dysfunction seen in ALS and CDM. To date, over 160 different point mutations in the SOD1 gene have been identified in ALS patients [11], with approximately 20% of all familial cases and 1-2% of sporadic cases due to mutations in this gene [7, 12].

A previous study by Awano and colleagues [13] identified a mutation in the canine SOD1 gene (*SOD1:c.118G > A*), where a G to A substitution in exon 2 causes an E40K missense mutation. Homozygosity for the A allele was strongly associated with CDM in the five breeds examined (Pembroke Welsh Corgi, Boxer, Rhodesian ridgeback, GSD and Chesapeake Bay retriever), where 96% of dogs diagnosed with CDM were A/A homozygous compared to 34% in the control dogs. In their North American population of GSDs, 4 out of 5 affected dogs were homozygous for the mutant A allele, compared to only 7 of 120 control GSDs, suggesting that genotyping for this mutation in GSDs might provide important diagnostic information. Therefore, the aim of the present study was to develop a simple genotyping test for the SOD1:c.118G > A mutation and to estimate the prevalence of this mutation in a UK population of GSDs.

## Results

The aim of this study was to develop a simple genotyping test for the *SOD1:c.118G > A* mutation using PCR followed by Restriction Fragment Length Polymorphism (RFLP). The G to A change at position 118 caused by the mutation alters a restriction digestion site, recognised by the Eco571 enzyme (CT**G**AAG(N)_16_), such that the DNA from the wild type (G) allele can be cut but that from the mutant (A) allele cannot.

**Figure 1:**
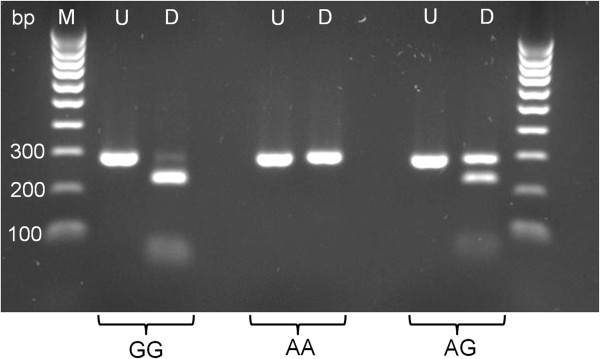
**RFLP analysis of three GSD, previously genotyped for** ***SOD1:c.118G > A*** **. M = DNA ladder with relevant sized bands indicated, U = undigested PCR product, D = PCR product digested with Eco571.** The digested G/G genotype shows bands at 230 bp and 62 bp, no digestion has occurred with the A/A genotype, and with the digested heterozygous A/G genotype, three bands are visible at 292 bp, 230 bp and 62 bp.

Genomic DNA from two GSDs of each SOD1 genotype, as determined by direct sequencing, was initially used to evaluate the RFLP assay. The initial PCR reaction produces a single amplicon of 292 bp which, after digestion, yields a distinct pattern of fragments, depending on the SOD1 genotype (Figure 1). In dogs with the homozygous G/G genotype, bands at 230 bp and 62 bp are present after digestion, whereas in the homozygous A/A mutant genotype, no digestion occurs and the original band at 292 bp remains. In the heterozygous G/A genotype, three bands are seen at 292 bp, 230 bp and 62 bp. Incomplete digestion of the PCR product in homozygous G/G dogs sometimes resulted in a feint band being visible at 292 bp after digestion, but this could be clearly distinguished from the more intense band representing the undigested fragment obtained after digestion in heterozygous G/A dogs.

One hundred and fifty GSDs, referred to a veterinary tertiary hospital in the UK between 2005 and 2010, were genotyped for the SOD1:c.118G > A mutation. The study population consisted of 50 dogs aged less than 6 years (i.e. before any clinical signs of CDM would be anticipated), 50 dogs over 8 years of age referred to the hospital neurology service with pelvic limb ataxia as a major clinical sign and 50 dogs over 8 years of age, referred to other clinical services with no neurological disease reported.

**Figure 2:**
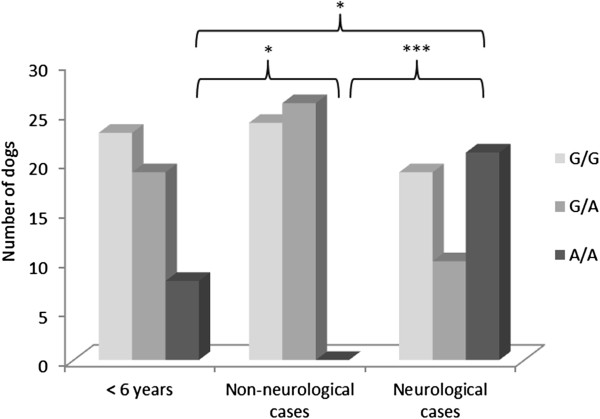
**Prevalence of SOD1:c.118G > A in a UK population of GSDs.** GSDs were grouped according to their age/clinical status and SOD1 genotype. Statistical analysis was undertaken using the Fishers Exact Test with a Bonferroni correction to compare the proportion of dogs homozygous for the mutation in each of the groups (*p < 0.05, ***p < 0.001).

The number of dogs displaying each of the SOD1 genotypes was found to vary in the three populations of GSDs examined (Figure 2). In the population of GSDs less than 6 years of age, 8 were homozygous mutant (A/A), 19 were heterozygous (G/A) and 23 dogs were homozygous wild type (G/G), which relates to an allele frequency of 0.35 for the *SOD1:c.118A* mutation in this GSD population. In the population of GSDs older than 8 years of age with non-neurological conditions, no dogs were homozygous (A/A), 26 were heterozygous (G/A) and 24 were homozygous (G/G), which gives an allele frequency 0.26 for the mutation. The frequency of dogs homozygous for the A allele was significantly lower in the dogs with non-neurological conditions compared to the young GSD population (p = 0.017). In the neurology case population of GSDs older than 8 years of age, 21 were homozygous (A/A), 10 were heterozygous (G/A) and 19 dogs were homozygous (G/G), which gives an allele frequency of 0.52 for the mutation. The frequency of dogs homozygous for the A allele in the ataxic case population is significantly higher than in both the young GSD population (p = 0.023) and the non-neurological case population (p < 0.001). However, when the results for GSDs over 8 years of age are combined (i.e. both ataxic and non-neurological cases) and compared with the young GSDs, there were no significant differences in allele or genotype frequencies. The allele frequency for the *SOD1:c.118A* mutation overall was 0.38.

## Discussion

The *SOD1:c.118G > A* mutation, associated with CDM, was originally identified in a genome-wide association study and characterised by sequence-based typing [13]. Recent studies have used conventional and real-time PCR techniques to develop more rapid and cost-effective methods of genotyping dogs for this particular mutation [14–16]. The present study employed PCR followed by RFLP analysis, utilising the Eco571 enzyme, demonstrating that this technique allows rapid and simple genotyping dogs for the *SOD1:c.118G > A* mutation.

There was no significant difference in allele or genotype frequencies comparing young GSDs (n = 50) with those over 8 years of age (n = 100) in the sample population from a large tertiary veterinary hospital. In dogs over 8 years of age, homozygosity for the *SOD1:c.118A* allele was strongly associated with those affected with pelvic limb ataxia, compared to those referred with non-neurological disease. None of the 50 dogs with non-neurological conditions were of the A/A genotype, compared with 21 of 50 dogs with neurological signs being homozygous for this mutation. This is a lower proportion of homozygous A/A mutant dogs than that identified in the American GSD CDM case population examined by Awano and colleagues [13], where 4 of 5 dogs were homozygous for the A allele, but similar to that found in an Italian study, where 3 of 10 GSD in their case population were homozygous A/A [15]. This might represent genetic variability, due to geographical differences in the gene pool of GSD populations studied, but more likely results from differences in selection criteria used for identifying the case populations. In both the current and the Italian study, selection of the case population was based on a broad phenotype, relating to neurological signs (such lower limb ataxia in this study), compared with the study of dogs in the USA, where a more definite diagnosis of CDM was made following post-mortem histopathology or MRI studies. Thus, although CDM cases would be included in our case population, this is a relatively heterogeneous group, with other causes of pelvic limb ataxia also likely to be represented.

Use of SOD1 genotyping in a clinical setting is now considered to be a useful component of the diagnostic panel, with dogs showing consistent clinical signs and being homozygous for the mutation, raising the index of suspicion of CDM, whereas those with heterozygous or homozygous wild-type genotypes considered more likely to be affected by other disease processes. As management of CDM is problematic, with the disease often rapidly progressing, the results of such a genetic test may influence the treatment the dog receives, particularly in those dog with co-morbidities, whereby it may not be appropriate to consider surgical intervention for concurrent spinal conditions, in a dog that is homozygous for the *SOD1:c.118G > A* mutation, but to opt instead for physiotherapy which has been shown to prolong life in dogs with CDM [17].

Although CDM appears to be inherited in an autosomal recessive manner, with incomplete penetrance [13], in our study of UK GSDs, all dogs over 8 years of age that expressed the A/A genotype were showing clinical signs of ataxia, with none of the ‘neurologically healthy’ dogs expressing this genotype. This suggests that, in the UK at least, penetrance is relatively high if not complete.

We also assessed the prevalence of the *SOD1:c.118G > A* mutation in a cross-sectional study of relatively young GSDs, which would be unbiased with respect to CDM susceptibility because they are too young to show clinical signs. The prevalence of the *SOD1:c.118A* allele in this population was estimated to be 0.35, with 8 of 50 dogs (16%) being homozygous for the A allele. Other studies [13, 15] using limited numbers of dogs have estimated the prevalence of the *SOD1:c.118A* allele in GSDs to be 0.17-0.21 with 4–6% being AA homozygous. However, these GSD populations were from different geographical locations and included dogs aged 0 to 14 years with and without evidence of neurological disease, and therefore do not necessarily represent the GSD population as a whole. A much larger study [16], where over 6000 GSDs were genotyped for the *SOD1:c.118G > A* mutation, revealed a frequency of 0.37 for the *SOD1:c.118A* allele with 22% being AA homozygotes, which is similar to the results from the present study. Follow up on the eight A/A homozygous dogs identified in the present study revealed that one dog had been euthanased due to clinical signs consistent with CDM, four dogs had been euthanased before reaching 7 years of age for other conditions such as neoplasia and liver disease, and three dogs currently remain disease free at 8 years of age, although they might still develop CDM in the future.

It has been suggested that genetic screening of dogs for the *SOD1:c.118G > A* mutation in breeds known to be susceptible to CDM might be used to influence breeding programs [3]. Genetic testing and identification of heterozygous or homozygous mutant dogs, alongside a strategy for ensuring that they are only bred to homozygous wild-type dogs, would reduce the prevalence of the SOD1 mutation in the population over several generations. A more radical approach, based on selection against all carriers of the mutation for breeding purposes, could pose a problem in terms of restricting genetic diversity, particularly in a breed such as the GSD, where the prevalence of the A allele is relatively high. In breeds such as the Boxer and Pembroke Welsh Corgi, where the prevalence of the A allele is even higher (A allele frequency is estimated to be over 0.7 in some studies [13]), breeding programs to select against dogs carrying the mutant A allele would likely have a major impact on the overall genetic health of these breeds, thus eliminating desirable qualities or unintentionally selecting for other genetic mutations.

## Conclusions

We have found the RFLP assay for the canine *SOD1:c.118G > A* mutation to allow rapid and cost-effective genotyping of GSD. Since no definitive ante-mortem test is available for CDM, this simple genetic test provides useful information to the clinician, faced with a dog demonstrating clinical signs where CDM is a differential diagnosis. This is particularly valuable for treatment planning and prognostication, especially when neurological/musculoskeletal co-morbidities exist. Careful consideration should be given when recommending more wide ranging genetic testing of younger GSD for selection of dogs for breeding, since there is a relatively high allele frequency (estimated at 0.35 in the UK) and proportion of carriers in the population. A more measured approach to reducing the prevalence of this particular mutation in the GSD population by avoiding breeding of carriers/affected dogs with those of a similar genotype would be recommended.

## Methods

EDTA blood samples were obtained from the genetic archive of the Royal Veterinary College, University of London. Samples were from GSD (n = 150) that had been referred to the Queen Mother Hospital for Animals between 2005 and 2010 and had been archived following completion of diagnostic testing and with informed owner consent for their use in clinical research. The samples selected consisted of 50 dogs <6 years of age, 50 dogs >8 years of age, referred for neurological assessment and with pelvic limb ataxia as a major clinical sign and 50 dogs >8 years of age referred to other clinical services with no neurological disease reported.

Genomic DNA was extracted from EDTA blood using the GenElute Blood Genomic Kit (Sigma-Aldrich) according to the manufacturer’s instructions. The isolated DNA was then used in PCR to amplify a region of the canine SOD1 gene. Primers (Sigma-Aldrich) were designed that flank the mutation region of the SOD-1 gene to generate a 292 base pair (bp) amplicon. The primers used were sense: 5′-AGTGGGCCTGTTGTGGTATC-3′ and antisense: 5′-TCTTCCCTTTCCTTTCCACA-3′. The PCR was performed in 25 μl reaction volumes using Immolase DNA polymerase (Bioline), with 2 μl genomic DNA as template and 2 μl of 10 pmol/μl primers. Thermocycling conditions consisted of an initial polymerase activation at 95°C for 10 min, followed by 37 cycles of 94°C for 40 s (denaturation), 55°C for 30 s (annealing) and 72°C for 1 min (elongation) with a final extention step of 72°C for 10 min (G-Storm GS1 thermal cycler, GRI).

The PCR products were purified using the GenElute PCR Clean-up Kit (Sigma-Aldrich) and then digested using Eco571 (Fermentas) for 1 h at 37°C. Digested PCR products were analysed by 2% agarose gel electrophoresis containing 6% Safe View Nucleic acid Stain (NBS Biologicals Ltd.) and using a 100 bp molecular weight ladder (Hyperladder IV, Bioline) to determine product sizes. The gels were visualised under UV light (ImageMaster VDS, Pharmacia Biotech/GE Healthcare).

The proportion of homozygous mutants in each group (n = 3) was compared using the Fisher’s exact test with a Bonferroni correction for multiple comparisons.

## Abbreviations

ALS: Amyotrophic Lateral Sclerosis
CDM: Canine Degenerative Myelopathy
GSD: German Shepherd Dog
RFLP: Restriction Fragment Length Polymorphism
SOD1: Superoxide Dismutase 1.
